# Diagnosis of infant synostotic and nonsynostotic cranial deformities: a review for pediatricians

**DOI:** 10.1016/j.rppede.2016.02.005

**Published:** 2016

**Authors:** Enrico Ghizoni, Rafael Denadai, Cesar Augusto Raposo-Amaral, Andrei Fernandes Joaquim, Helder Tedeschi, Cassio Eduardo Raposo-Amaral

**Affiliations:** a Universidade Estadual de Campinas (Unicamp), Campinas, SP, Brazil; b Instituto de Cirurgia e Plástica Crânio Facial, Hospital SOBRAPAR, Campinas, SP, Brazil

**Keywords:** Craniofacial abnormalities, Craniosynostosis, Diagnosis, Pediatricians

## Abstract

**Objective:**

To review the current comprehensive care for nonsyndromic craniosynostosis and nonsynostotic cranial deformity and to offer an overall view of these craniofacial conditions.

**Data source:**

The review was conducted in the PubMed, SciELO, and LILACS databases without time or language restrictions. Relevant articles were selected for the review.

**Data synthesis:**

We included the anatomy and physiology of normal skull development of children, discussing nuances related to nomenclature, epidemiology, etiology, and treatment of the most common forms of nonsyndromic craniosynostosis. The clinical criteria for the differential diagnosis between positional deformities and nonsyndromic craniosynostosis were also discussed, giving to the pediatrician subsidies for a quick and safe clinical diagnosis. If positional deformity is accurately diagnosed, it can be treated successfully with behavior modification. Diagnostic doubts and craniosynostosis patients should be referred straightaway to a multidisciplinary craniofacial center.

**Conclusions:**

Pediatricians are in the forefront of the diagnosis of patients with cranial deformities. Thus, it is of paramount importance that they recognize subtle cranial deformities as it may be related to premature fusion of cranial sutures.

## Resumo

**Objetivo:**

Revisar o atendimento integral atual de craniossinostose não sindrômica e deformidade craniana não sinostótica e oferecer uma visão global dessas condições craniofaciais.

**Fontes de dados:**

A revisão foi feita nas bases de dados PubMed, SciELO e Lilacs e sem restrições de tempo ou idioma. Artigos relevantes foram selecionados para a revisão.

**Síntese dos dados:**

Foram incluídas a anatomia e a fisiologia do desenvolvimento normal do crânio em crianças, discutidas nuances relacionadas à nomenclatura, epidemiologia, etiologia e ao tratamento das formas mais comuns de craniossinostose não sindrômica. Também foram discutidos os critérios clínicos para o diagnóstico diferencial entre deformidades posicionais e craniossinostose não sindrômica. Deram-se aos pediatras subsídios para um diagnóstico clínico rápido e seguro. Se deformidades posicionais forem diagnosticadas com precisão, elas podem ser tratadas com sucesso por meio da modificação do comportamento. Dúvidas de diagnóstico e pacientes portadores de craniossinostose devem ser encaminhados imediatamente a um centro multidisciplinar craniofacial.

**Conclusões:**

Os pediatras estão na vanguarda do diagnóstico de pacientes com deformidades cranianas. Assim, é de suma importância que reconheçam deformidades cranianas sutis, pois elas podem estar relacionadas à fusão prematura das suturas cranianas.

## Introduction

Cranial deformities are a common complaint in pediatric units, since 25% of infants of single pregnancies and 50% of multiple pregnancies have some degree of skull deformity at birth. In general, parents usually recognize these changes in the first weeks or months of life.^1^ However, in some scenarios the diagnosis may be overlooked by the family that tends to deny the problem. In these cases, pediatricians must be aware of these issues and counsel the family for the importance to seek a craniofacial team. In addition, it is of fundamental importance that pediatrician be prepared at first consultation to make the differential diagnosis between a positional deformity and craniosynostosis, considering that children born with a positional deformity does not need to be exposed to the ionizing radiation of a computed tomography (CT), apart from the costs of the procedure and the sedation risks to achieve it.^2^

In this report, we review the anatomy and physiology of normal skull development of children, discussing nuances related to nomenclature, epidemiology, etiology, and treatment of the most common forms of craniosynostosis. The clinical criteria for the differential diagnosis between positional deformities and craniosynostosis are also presented, allowing the pediatrician subsidies for a quick and safe clinical diagnosis.

## Method

The present study is a literature review, with a descriptive approach. We performed a literature review by searching the Medline (PubMed), SciELO, and LILACS databases without time or language restrictions. The final literature review was performed on July 2015. To identify all relevant articles (review articles, clinical trials, and cohort studies) about the current comprehensive care for nonsyndromic craniosynostosis and nonsynostotic cranial deformity the following search terms were used: “nonsyndromic craniosynostosis”, “nonsynostotic cranial deformity”, “positional deformity”. “nonsynostotic posterior plagiocephaly”, and “positional plagiocephaly”. Each relevant study was individually reviewed to identify information concerning normal skull development of children, nomenclature, epidemiology, etiology, diagnosis, and treatment of the most common forms of nonsyndromic craniosynostosis and nonsynostotic cranial deformity.

### Cranial anatomy

The skull of a newborn is composed of multiple bones and sutures that make it malleable and subject to external forces that deform it, to enable its passage through the birth canal and to accommodate the encephalon, since the brain volume is quadrupled in the first two years of life.^3^

The skull is composed of four major sutures (metopic, sagittal, coronal, and lambdoid), three secondary sutures (frontonasal, temporal squamosal, and frontosphenoidal), and four main bones (temporal, frontal, parietal, and occipital). The metopic suture separates the frontal bones from each other, the sagittal suture from the parietal bones; the coronal suture from the parietal and frontal bones, and the lambdoid suture from the parietal and occipital bones.^3^ Besides the bones and sutures, soft and membranous space separating the skull bones are named fontanelle, being of great importance are the anterior or bregmatic (bounded by the frontal and parietal bones) and the posterior or lambdoid (bounded by the occipital bone and parietal bones).

Under physiological conditions, the cranial sutures progress into fusion with different initial periods of fusion according to each major suture (Table 1). The same occurs with the fontanelles, which usually close themselves by the second year (Table 2).

**Table 1:** Major and secondary skull sutures and age at the onset of fusion^3^

Sutures	Beginning of fusion
Metopic	2 months
Sagittal	22 months
Coronal	24 months
Lambdoid	26 months
Frontonasal	68 months
Frontosphenoidal	22 months
Temporal squamosal	35-39 months

**Table 2:** Age of closure of cranial fontanelles^3^

Fontanelles	Age of closure
Anterior or bregmatic	24 months
Posterior or lambdoid	3 months
Anterolateral (Sphenoid)	6-24 months
Posterolateral (Mastoid)	6-24 months

An important detail is that the skull bones are membranous (without a prior cartilaginous phase), which results in growth through the bone deposit in the region of the sutures; this growth occurs perpendicular to the suture. For example, coronal sutures allow the anterior-posterior growth of the skull, while the sagittal suture allows growth of the biparietal skull.^3^

### Craniosynostosis

The definition of craniosynostosis is the premature fusion of one or more cranial sutures. The occurrence is approximately one for 2000 to 2500 live births.^4^ The premature fusion of sutures prevents perpendicular growth of the skull, and an increase in brain volume leads to a compensatory growth of the skull parallel to this.

**Figure 1:**
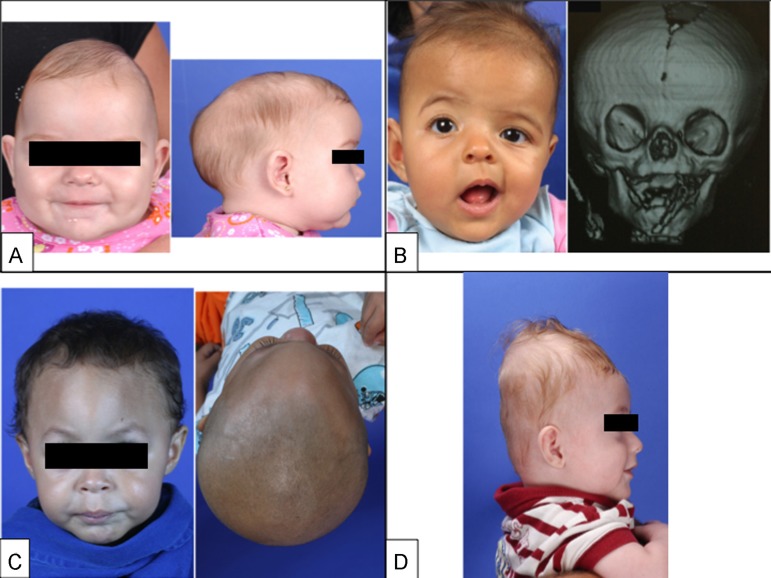
(A, Left) Frontal photograph of patient with premature fusion of sagittal suture showing the characteristic temporal pinching. (Right) Lateral photograph reveling increase in the anterior-posterior diameter of the skull (long narrow skull), the frontal bossing and occipital bulging (occipital bullet), which are the main clinical characteristics of sagittal craniosynostosis. (B, Left) Frontal photograph of patient with premature fusion of the right coronal suture showing the retrusion of the ipsilateral frontal bone fusion and compensatory contralateral bulging, asymmetry of the eyebrows, orbits, ears, nose, jaw, as well as convergent strabismus of the left eye. (Right) 3D CT reconstruction showing the premature fusion of the right coronal suture and the elevation of the ipsilateral sphenoid wing leading to an elongate orbit, recognized as the “harlequin orbit”. (C, Left) Frontal photograph of patient with premature fusion of metopic suture showing the triangular aspect of the forehead with retruded crests of the orbits bilaterally and hypoteleorbitism (approximation of orbits). (Right) Basal view revealing the triangular appearance of the skull. (D) Lateral photograph of patient with premature fusion of lambdoid suture showing the turricephalic aspect of the skull. Two-dimensional photographs and radiological documentations belong to SOBRAPAR Hospital's archives. Informed consent forms were signed by the patient's parents.

Craniosynostosis are classified as primary or secondary. Primary craniosynostosis results from genetic and environmental influences, being classified as simple and complex. Complex craniosynostosis are divided even further into nonsyndromic and syndromic (Table 3).^5^^-^ 8 Due to the greater prevalence, we will discuss the diagnosis of simple primary craniosynostosis, namely sagittal, coronal, metopic, and lambdoid synostoses (Fig. 1). Then the nonsynostotic posterior plagiocephaly (positional plagiocephaly) will be presented, emphasizing important features for differential diagnosis with lambdoid synostosis (posterior plagiocephaly).

**Table 3:** Classification of craniosynostoses.

1. *Primary*
Simple (involving a single suture): Sagittal, Coronal, Metopic, and Lambdoid
Complex (fusion of two or more sutures)
Nonsyndromic: Bicoronal
Syndromic: Crouzon, Apert, Pfeiffer, and Saethre-Chotzen

2. *Secondary*
Metabolic disorders: Hyperthyroidism, Inborn Errors of Metabolism
Various malformations: Microcephaly, Encephalocele
After ventricular shunt with excessive drainage of CSF (cerebrospinal fluid)
Fetal exposure to certain substances: Valproic acid, Phenytoin
Mucopolysaccharidosis

### Genetic aspects

Multiple hypotheses have been proposed to explain the pathogenesis of abnormal suture fusion. Both environmental (especially intrauterine fetal head constraint) and genetics factors (single gene mutations, chromosome abnormalities and polygenic background) predispose to craniosynostosis.^9^^-^ 11 Mutations in 7 genes (namely, FGFR1, FGFR2, FGFR3, TWIST1, EFNB1, MSX2 and RAB23) are unequivocally associated with Mendelian forms of syndromic craniosynostosis.^9^^-^ 11 In contrast, the genetic etiology of nonsyndromic craniosynostosis remained poorly understood until very recently.^9^^-^ 11 In the last years, epidemiologic and phenotypic studies clearly demonstrate that nonsyndromic craniosynostosis is a complex and heterogeneous condition supported by a strong genetic component accompanied by environmental factors that contribute to the pathogenesis network of this birth defect.^9^^,^ 10 In fact, rare mutations in FGFRs, TWIST1, LRIT3, ALX4, IGFR1, EFNA4, RUNX2, and FREM1 have been reported in a minor fraction of patients with nonsyndromic craniosynostosis.^9^^,^ 10 The minimum molecular genetic tests recommended for each clinical presentation (syndromic and nonsyndromic craniosynostosis) have been previously published review^10^ and are out of the scope of this report. Further research of a large population with phenotypically homogeneous subsets of patients is required to understand the complex genetic, maternal, environmental, and stochastic factors contributing to nonsyndromic craniosynostosis.^9^^-^ 11

### General diagnostic approach

Pediatricians are expected to be able to recognize skull deformities and to diagnose them as either craniosynostosis or a positional skull deformity. If a skull deformity is present, the physical examination and clinical history (key features described in the next subheads) are the most helpful and revealing pieces of information in the child's evaluation. A previous published anamnestic flowchart^12^ serves as a guideline to distinguish craniosynostosis from positional skull deformities. The key questions to differentiate the craniosynostoses from the nonsynostotic deformities are: (1) “Is deformity present at birth?” Craniosynostosis is present at birth, whereas nonsynostotic deformities develop in the neonatal period; (2) Is there a preferred sleep position?; (3) “Is there improvement of the deformity?” Craniosynostosis gets worse with time, whereas the nonsynostotic deformities improve as the child develops head control and the skull no longer has localized pressure for long periods”.^12^

In rare and difficult cases, when the examination and history are not diagnostic, a good-quality four-view radiographic series (anteroposterior, Towne and two lateral projections) might be sufficient to exclude craniosynostosis and avoid further radiation exposure.^13^ If it is unclear, because of the very young age of the patient, it is recommended to repeat X-skull after 1 or 2 months.^14^ CT is not the recommended modality for screening because of the associated radiation exposure and high imaging costs and diagnostics by pediatricians with CT is associated with further delay in referral.^13^^,^ 14

After the clinical suspicion (or confirmation) of craniosynostosis, the children should be referred to a multidisciplinary team specialized in craniofacial anomalies (anesthesiologist, plastic surgeon, speech therapist, neurosurgeon, orthodontist, otorhinolaryngologist, and psychologist).^15^ In these centers, the radiological exam of choice is the three-dimensional CT scan that contributes to elucidation of the extension of suture fusion and the consequent craniofacial deformity and subsequent surgical planning. It is noteworthy that the cephalic perimeter generally does not change due to compensatory growth of other bones in the majority of cases with simple craniosynostosis.

In this context, patients with craniosynostosis not surgically-treated can develop several complications such as^16^^-^ 18: Intracranial hypertension (ICH) occurs in up to 60% of children with complex craniosynostosis and 20% of carriers of simple craniosynostosis; cognitive and developmental disorders, poor weight gain, visual, hearing, and language disorders; and psychological problems such as low self-esteem and social isolation. Therefore, the objective of surgical treatment is to prevent ICH and to correct craniofacial abnormalities. Overall, the optimal timing of surgical correction in most cases is between 6 and 9 months of age. The motivations for performing the surgery before 1 year of age include the ability of the child younger than 1 year to completely reossify, the malleable character of the calvaria during this age, and the tremendous brain growth that occurs during the first year, which allows good remodeling of the skull.^19^ Satisfactory craniofacial form and esthetic pleasing outcomes have also been associated with craniofacial surgical interventions performed before 1 year of age.^18^^,^ 19 It is noteworthy that the presence of ICH signs (irritability, swelling of the papilla, bulging fontanelle, and imaging findings) may result in the need for earlier surgical intervention, to perform decompression procedures or ventricular shunt surgery if associated with hydrocephalus.

### Sagittal synostosis

It is the most common form of simple craniosynostosis and accounts for 40-60% of cases, being more prevalent among males (75-85%). The skull has an elongated and narrow shape, similar to a boat, hence being called scaphocephaly (Fig. 1A).^20^ Upon physical examination, a ridge can be palpated on the sagittal suture. It should be noted that the anterior fontanelle may not be closed. Compensatory frontal bossing and occipital protrusion may occur in varying degrees.

Surgical treatment is indicated between 3 and 12 months of age, and procedures may vary from a simple endoscopic resection of the sagittal suture to total reconstruction of the skull, depending on the severity of the clinical presentation. In our service, we recommend surgery between 6 and 9 months of age and use the craniectomy in a “π” fashion (named Hung Spun procedure),^21^ associated with several osteotomies (bone cuts) parallel rectangles of approximately two centimeters long in the parietal bone, between the coronal and lambdoid sutures, which permit greater lateral space for further accommodation of the brain. Barrel stave osteotomies (lateral bone cuts) still allow for reduction of the anterior posterior diameter and better remodeling of the skull, with excellent esthetic results.^22^

### Coronal synostosis

It is the second most common form, accounting for up to 25% of craniosynostosis cases. The closure of a coronal suture is called anterior plagiocephaly,^23^^,^ 24 while the closure of two sutures is termed brachycephaly (commonly found in syndromic craniosynostosis).^6^^-^ 8 It predominantly affects females (60%), with similar incidence on both sides.

Early fusion of the coronal suture leads to a flattening of the frontal bone and the ipsilateral orbital rim to the fusion, with a compensatory contralateral frontal bossing.^23^^,^ 24 Strabismus is a common finding (50-60% of cases) and is the result of morphological changes in the orbital roof and trochlea, altering the function of the superior oblique muscle.^25^ Elevation of the ipsilateral sphenoid wing can be seen in simple skull radiography and is recognized as the “harlequin orbit” (Fig. 1B).^26^ Premature fusion of the coronal suture also causes a deviation of the skull base, changing the position of the orbits, asymmetry of the eyebrows, asymmetry of the ear position, deviation of the mandible, and change of occlusion, with an important esthetic effect.^23^^,^ 24

Therefore, surgical treatment is indicated for correction of the morphological skull deformity and its repercussions on the face, but also because of strabismus and risks of developing ICH. We recommend the procedure between 6 and 9 months of age, when there is already sufficient bone maturity for remodeling. Basically, a front-orbital advancement associated with frontal remodeling is performed and releasing both coronal sutures.^24^ Further procedures might be necessary as fat injection in the craniofacial skeleton to decrease facial asymmetries and correction of eyelid ptosis. At the end of facial growth orthognatic surgery and rhinoplasty with septoplasty may be performed by a plastic surgeon with a craniofacial training.

### Metopic synostosis

Corresponds to 10% of all craniosynostosis and predominates in males (75-85% of cases). Early fusion of the metopic suture restricts the transversal growth of frontal bones, and in more severe cases can restrict the expansion of the anterior fossa, which leads to hypoteleorbitism, and consequently to trigonocephaly (Fig. 1C). Metopic craniosynostosis is the single suture synostosis most frequently associated with more cognitive disorders, primarily due to the growth restriction of the frontal lobes.^27^

The increase in the anterior fossa volume is the main objective in the treatment of patients with trigonocephaly, as well as frontal remodeling and fronto-orbital advancement. The best time for treatment is between 6 and 9 months of age.

### Lamboid synostosis

This is the rarest form of simple craniosynostosis, with an incidence of about 0.3 per 10,000 live births, corresponding to approximately 1.0-5.5% of all craniosynostosis. When evaluated in a population of children with occipital flattening (also called posterior plagiocephaly), it is responsible for only 0.9-4.0% of the cases.^28^

Fusion of a lambdoid suture leads to an occipital deformity (posterior plagiocephaly) with a trapezoidal shape, while the fusion of both lambdoid sutures leads to brachycephaly (Fig. 1D). In addition to posterior deformity (flattening, poor positioning of ears, parietal compensatory bossing) caused by premature fusion of the lambdoid suture, significant morphological changes may occur concomitantly in the posterior fossa. This craniosynostosis is associated with herniated cerebellar tonsils (also known as Chiari malformation type I) and fusion of the jugular foramen, resulting in a high risk of venous hypertension. Thus, this is the form of simple craniosynostosis with the greatest risk of ICH.^29^

Surgical treatment is based on volume expansion of the posterior portion of the skull (parietal and occipital region) and releasing the lambdoid sutures. However, this region has large venous drainage, with innumerable scalp veins that cross the bone toward the dural sinuses, greatly increasing the surgical risk of a craniotomy and bone remodeling. In our service, we choose a posterior distraction technique, in which the cranial volume is gradually increased, significantly reducing the risk of bleeding and need for blood transfusions.^30^

### Positional plagiocephaly

The term plagiocephaly means oblique skull and corresponds to a unilateral or bilateral occipital flattening, which may arise due to the continual influence of external forces on the immature skull (nonsynostotic posterior plagiocephaly) or because of premature fusion of one or both lambdoid sutures (synostotic posterior plagiocephaly). Anterior plagiocephaly can be used to define the cranial deformation characterized by premature unilateral fusion of the coronal suture.

Positional or deformational plagiocephaly is the most common cause of plagiocephaly (prevalence of 5-48% in healthy newborn infants)^31^ versus an incidence of 0.003% of synostotic plagiocephaly (lamboid synostosis).^28^

Based on the introduction of the campaign to prevent Sudden Infant Death Syndrome (“Back to Sleep”), in the beginning of the 1990s - which recommended that babies remain in the supine position - a significant increase in the incidence of children with positional plagiocephaly was noticed (5-48%).^31^ This deformity results from an ongoing action of gravitational forces on the occipital region, causing a flattened region of the posterior craniofacial skeleton. If no intervention is performed, the deformity can continue and, in severe cases, evolve with facial deformities. Positional plagiocephaly occurs more often on the right side (70% of cases) and affects more males. The major risk factors include: torticollis, prematurity, multiparity, and a fixed sleeping position.

Diagnosis is eminently clinical, and the differentiation with synostotic plagiocephaly (unilateral fusion of the lambdoid or coronal sutures) is essential.^28^ Anamnesis and physical examination are sufficient to establish the differential diagnosis between a positional deformity and craniosynostosis in the vast majority of cases (Table 4). Classically, patients with premature fusion of the lambdoid suture already have the deformity at birth, while those with a positional deformity have a normal skull at birth and develop the deformity in the subsequent weeks or months. When asked, parents may mention that there is a preferred position of the baby's positioning in patients with positional plagiocephaly, while in patients with synostotic plagiocephaly, there is no preferred position.

**Table 4:** Important characteristics to subsidize the differential diagnosis of positional plagiocephaly versus lamboid synostosis^21^^,^ 24 ^,^ 25

Characteristics	Positional plagiocephaly	Lambdoid craniosynostosis
Age at onset	Several weeks postnatally	Birth
Preferred position	Common	Rare
Torticollis	Present	Absent/Present
Bony ridge along the lambdoid suture	Absent	Present
Bulging mastoid	Absent	Present
Frontal bossing	Ipsilateral	Contralateral
Displacement of the ipsilateral ear	Anterior	Posterior
Skull shape	Parallelogram	Trapezoid
	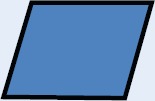	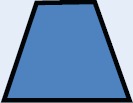
Diagnosis	Clinical, through medical history and physical examination	Three-dimensional computed tomography
Treatment	Clinical	Surgical

3D, three-dimensional; CT, computed tomography.

Pediatricians should perform a physical examination with the aid of the parents; initially, the patient remains on the mother/father's lap facing the pediatrician and after facing the parents; finally, the examiner should observe the child from a superior view. During the physical examination, symmetries between the skull, forehead, and ears should be carefully analyzed.

**Figure 2:**
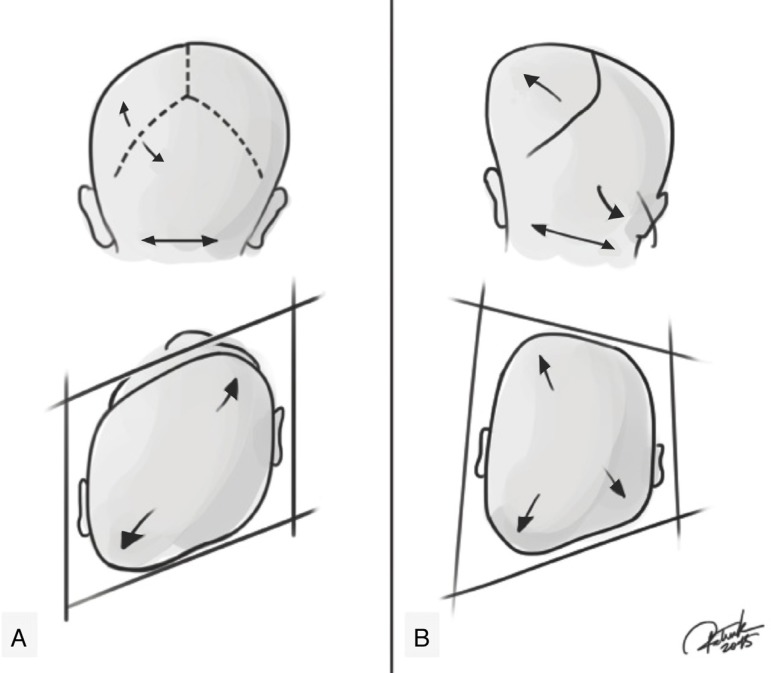
Representation of positional plagiocephaly and true (synostotic) posterior plagiocephaly. (A) Positional plagiocephaly showing: absence of lambdoid suture stenosis, format of a parallelogram skull, ipsilateral compensatory frontal bossing, ipsilateral ear in an anterior position, as if it had been pushed. (B) True posterior plagiocephaly showing: presence of lambdoid suture stenosis, shape of a trapezoid, ipsilateral bulging in the mastoid region, contralateral compensatory frontal bossing, ipsilateral ear stenosis tends to be in a posterior position and downwards, as if the suture pulled it. Credits: Patrick Braga.

Positional plagiocephaly presents a format of a parallelogram skull, while synostotic posterior plagiocephaly has the shape of a trapezoid.^32^ Still, an ipsilateral bulging in the mastoid region and a ridge on the fused lambdoid suture can be seen and palpated. In moderate to severe cases in both deformities, compensatory frontal bossing can be observed, ipsilateral in positional plagiocephaly and contralateral in synostotic plagiocephaly. This involvement of the forehead may progress, leading to a facial scoliosis in both pathologies, changing the facial symmetry. In patients with lambdoid suture craniosynostosis, the ipsilateral ear stenosis tends to be in a posterior position and downwards, as if the suture pulled it. While the positional plagiocephaly tends to be in an anterior position, as if it had been pushed (Fig. 2). The physical examination should also include evaluation of the cervical region and look for evidence of congenital torticollis and/or thickening of the sternocleidomastoid muscle, which are directly related to positional plagiocephaly.

The diagnosis and treatment of positional plagiocephaly are clinical.^32^ Some guidance should be given to parents at the first childcare consultation, how to avoid bad posture positions for sleeping or on the changing table, to note the presence of torticollis, to spend as little time as possible in the “baby comfort”, and to encourage that the baby stay for a longer time in the ventral decubitus position, while under supervision. It is important to diagnose any cervical restriction (e.g., congenital torticollis or thickening of the sternocleidomastoid muscle) and guide parents about the need of early physiotherapy treatment.

In addition to the guidelines, therapeutic measures can be employed, such as how to force the baby to sleep on the contralateral side of the deformity, to encourage moving the location of the crib and changing table, in order to force the baby to turn his head to the side he is on, besides stimulating the baby to sit. Such measures are effective until 4-6 months as treatment of positional plagiocephaly.^32^^,^ 33

The prevalence of positional deformity tends to decrease with age and may be as low as 3.3% at 2 years of age, which highlights the natural ability of skull remodeling.^32^ However, after 6 months of age, the use of a specific helmet may be indicated in patients with severe deformities to aid in remodeling the skull. It should be emphasized that the helmet requires at least 23h of use per day to be effective, which may result in pressure sores and local abrasions, besides the high cost of the appliance and bothersome to the child.^34^

## Conclusions

Cranial deformities are common complaints and highly prevalent in the routine of pediatricians. Although the vast majority of children present positional deformities, early diagnosis of craniosynostosis and referring them to specialized treatment in a timely manner is critical to optimize surgical outcomes. The diagnosis of positional deformities is usually clinical, and treatment consists of simple guidelines and measures to prevent worsening the condition. When there is a diagnosis of craniosynostosis, a multidisciplinary approach of the child with craniosynostosis is crucial for a greater surgical success rate and to minimize complications.
